# Event-related potential (ERP) evidence for visual processing differences in children and adults with cystinosis (CTNS gene mutations)

**DOI:** 10.1186/s13023-023-02985-y

**Published:** 2023-12-12

**Authors:** Douwe J. Horsthuis, Sophie Molholm, John J. Foxe, Ana A. Francisco

**Affiliations:** 1 The Cognitive Neurophysiology Laboratory, Department of Pediatrics, Albert Einstein College of Medicine, Van Etten Building, Suite 1C, 1225 Morris Park Avenue, Bronx, NY 10461 USA; 2 Department of Neuroscience, Rose F. Kennedy Center, Albert Einstein College of Medicine, Bronx, NY USA; 3 The Frederick J. and Marion A. Schindler Cognitive Neurophysiology Laboratory, Ernest J. Del Monte Institute for Neuroscience & Department of Neuroscience, University of Rochester School of Medicine and Dentistry, Rochester, NY USA

**Keywords:** EEG, Visual evoked potential, P1, Copy number variation, Lysosomal storage disorder

## Abstract

**Background:**

Cystinosis, a rare lysosomal storage disease caused by mutations in the CTNS gene, is characterized by cystine crystallization and accumulation within multiple tissues, including kidney and brain. Its impact on neural function appears mild relative to its effects on other organs during early disease, but since therapeutic advances have led to substantially increased life expectancy, neurological implications are of increasing interest, necessitating deeper understanding of the impact of cystinosis on neurocognitive function. Behavioral difficulties have been reported in cystinosis in the visual domain. Very little is known, however, about how the brains of people living with cystinosis process visual information. This is especially interesting given that cystine accumulation in the cornea and posterior ocular structures is a hallmark of cystinosis.

**Methods:**

Here, high-density scalp electrophysiology was recorded to visual stimuli (during a Go/No-Go task) to investigate visual processing in individuals with cystinosis, compared to age-matched controls. Analyses focused on early stages of cortical visual processing.

**Results:**

The groups differed in their initial cortical response, with individuals with cystinosis exhibiting a significantly larger visual evoked potential (VEP) in the 130–150 ms time window. The groups also differed in the associations between neural responses and verbal abilities: While controls with higher IQ scores presented larger neural responses, that relationship was not observed in cystinosis.

**Conclusions:**

The enlarged VEP in cystinosis could be the result of cortical hyperexcitability and/or differences in attentional engagement and explain, at least partially, the visual and visual-spatial difficulties described in this population.

**Supplementary Information:**

The online version contains supplementary material available at 10.1186/s13023-023-02985-y.

## Introduction

Cystinosis is an autosomal recessive disorder caused by bi-allelic mutations in the 17p13.2-located CTNS gene [1] with an incidence rate of around one in 100,000 to 200,000 live births [2, 3]. The accumulation of cystine in cells [4, 5], characteristic of the condition, leads to deregulation of endocytosis and cell signaling [6]. Ultimately, intralysosomal cystine crystallizes, triggering significant damage in a multitude of tissues and organs [7]. Renal, retinal, endocrinological, muscular, and neurological complications are observed [8, 9]. Due to advances in drug development and the availability of renal replacement therapy [10, 11], the life expectancy of individuals with cystinosis has, however, increased well into adulthood, demanding a better understanding of the developmental trajectories associated with the condition.

To reduce this gap in knowledge, our research team has focused on characterizing the impact of cystinosis on brain and cognitive function. Though abnormally high levels of cystine have been observed in different brain regions [12–14], cystinosis’ impact on brain activity is still not well understood. In previous work focusing on auditory processing and sensory memory, we reported generally maintained sensory processing, but some differences in sensory memory in children and adults living with cystinosis [15, 16].

Here, we expand this investigation to the visual sensory domain. Interestingly, visual-perceptual indices are often significantly lower than verbal indices in individuals with cystinosis [17–19]. This pattern emerges early in development and persists throughout the lifespan [20, 21], regardless of age at treatment onset [22]. Significant difficulties have also been described in domains related to visual-motor, visual-spatial and visual memory skills [22–26], but see [27] for a report of maintained visual learning in adults with cystinosis. Despite this pattern of relative weaknesses in visual processing domains, very little is known about how the brains of people living with cystinosis process visual information. One case study tested visual processing in two children with cystinosis before and after kidney transplantation. During dialysis treatment, both children showed delayed and decreased visual evoked responses, but typical brain responses were seen after kidney transplantation [28]. Despite the very small number of individuals tested to date, EEG measurements appear to be sensitive to neuropathology in cystinosis and could be useful as outcome measures to assess the impact of treatment on brain function.

Here, we use high-density EEG to investigate basic visual processing in a group of individuals with cystinosis and compare them to a group of age-matched controls. The analyses focus on the visual evoked potential (VEP) component P1. P1 is an early VEP peaking around 100 ms following stimulus onset and has been associated with multiple generators in both dorsal and ventral visual streams [29–33]. Additionally, we tested for associations between neural responses and age and standardized cognitive measures.

Considering the behavioral difficulties reported in visual-related processing in cystinosis and the tendency for cystine accumulation in the retina, we hypothesized that individuals with cystinosis would show different sensory-perceptual brain responses to visually presented stimuli, reflected in amplitude differences in the P1 component of the VEP. Identifying the processing stages that are impaired and contribute to visual and visual-spatial processing differences in cystinosis will be important in the development of impactful strategies that address visual processing difficulties and identify sensitive biomarkers of treatment efficacy on brain function.

## Materials and methods

### Participants

Thirty-eight individuals diagnosed with cystinosis (CYS; age range: 7–36 years old, 25 women) and 45 age-matched controls (CT; 27 women) were recruited. Individuals with cystinosis were recruited via social media and through contact with family organizations. Due to the rareness of cystinosis, most participants, all of whom lived within the United States, traveled from out-of-state to participate. Furthermore, the controls were recruited via flyers in the neighborhoods surrounding the lab and through a lab-maintained participant database. Developmental and/or educational difficulties or delays, neurological problems, and a severe mental illness diagnosis were exclusionary criteria for controls. Current neurological problems were exclusionary criteria for individuals living with cystinosis. To assess visual acuity, a Snellen chart was used. All participants had normal or corrected to normal vision. Nevertheless, all participants were asked at the start of the electrophysiological experimental session if they could see the stimuli and their different components without difficulty. One individual with cystinosis was excluded from the final sample due to illness on the scheduled day of testing. All individuals, and their legal guardian if under 18 years old, signed a consent form. Participants were monetarily compensated for their time. This study and all the associated procedures were approved by the Albert Einstein College of Medicine Institutional Review Board (IRB 2009-523). All aspects of the research conformed to the tenets of the Declaration of Helsinki.

### Experimental procedure and stimuli

Participation consisted of two visits, which involved completion of a cognitive function battery and EEG recordings. The cognitive function battery included the assessment of verbal and non-verbal intelligence (using age-appropriate Wechsler Intelligence Scales) and executive functioning components (Delis-Kaplan Executive Function System, D-KEFS; [34] and the Conners Continuous Performance Test 3, CPT; [35]). During the EEG recording session, participants were asked to respond to different tasks assessing sensory processing and response inhibition. Here, we focus on basic visual processing of images presented in the context of a Go/No-Go task. Response inhibition (cognitive function and EEG) findings are reported separately [36].

During the EEG Go/No-Go task, positive and neutral valence images from the International Affective Picture System (IAPS) were presented in a pseudorandom sequence. Participants were instructed to press the left mouse button with the right index finger upon each stimulus presentation, as quickly and as accurately as possible, unless the stimulus was a repetition of the immediately preceding stimulus, in which case they should withhold their response (i.e., not push the mouse button). Stimuli, subtended 8.6° horizontally by 6.5° vertically, were presented centrally for 600 ms at an average rate of 1 per second (every 950–1050 ms with a random temporal jitter within this 100 ms window). Three 12-min blocks were run. Each block consisted of 540 trials, for a total of 1620 per participant. Here, we focus exclusively on hit trials, that is, trials that included a correct button press after stimulus presentation (only hits preceded by another hit were included), to ensure a good signal-to-noise ratio and to avoid overlap of processes related to inhibiting a response (on withhold trials).

### Data acquisition and analysis

Continuous EEG data were recorded from 64 scalp electrodes at a sampling rate of 512 Hz (Active 2 system; Biosemi™, The Netherlands; 10–20 montage) and preprocessed using the EEGLAB toolbox (version 2021.0) [37] for MATLAB (version 2021a; MathWorks, Natick, MA) (the full pipeline can be accessed at: https://github.com/DouweHorsthuis) [38]. Preprocessing steps included down-sampling data to 256 Hz, re-referencing to the average, and filtering with a 0.1 Hz high pass filter (0.1 Hz transition bandwidth, filter order 16,896) and a 45 Hz low pass filter (11 Hz transition bandwidth, filter order 152). Both were zero-phase Hamming windowed sinc FIR filters. Noisy channels were excluded based on kurtosis and visual confirmation. Artifacts from blinks and saccades were eliminated via Independent Component Analysis (ICA). The spherical spline method was then used to interpolate channels that were removed in previous steps. Data were segmented into epochs of −100 ms to 400 ms using a baseline of −100 ms to 0 ms. These epochs went through an artifact detection algorithm (moving window peak-to-peak threshold at 120 µV). To equate number of epochs in each participants averaged VEP, 200 epochs meeting the criteria for hits were randomly selected per participant.

P1 was measured between 130 and 150 ms at O1, Oz, and O2. Time windows and electrode locations were selected based on past research and confirmed (and adjusted) by inspecting the timing and topography of the major voltage fluctuations in the grand averages. Mean amplitude data were used for both between-groups statistics and Spearman correlations. All *p*-values (from *t*-tests and Spearman correlations) were submitted to Holm-Bonferroni corrections for multiple comparisons [39], using the *p.adjust* of the *stats* package in R [40]. Mixed-effects models were implemented to analyze trial-by-trial data, using the *lmer* function in the *lme4* package [41] in R [40]. Group was always a fixed factor. Participants and trials were added as random factors. Models were fit using the maximum likelihood criterion. *p*-values were estimated using *Satterthwaite* approximations.

## Results

### Demographics and cognitive function measures

**Fig. 1:**
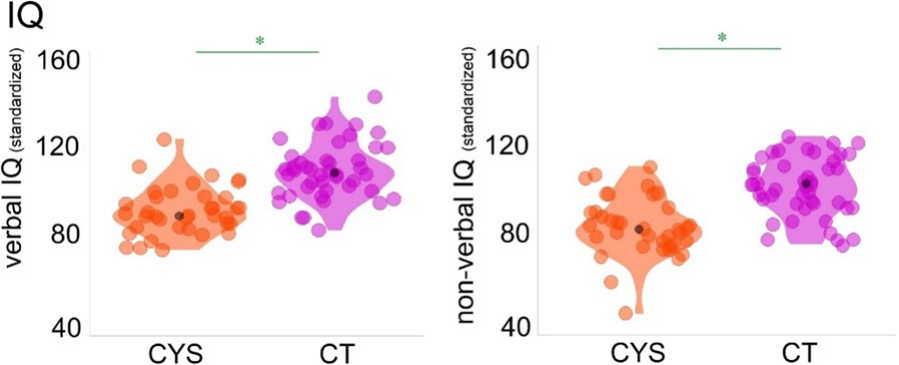
Included participants’ verbal and non-verbal IQ scores. Violin plots showing individual data-points (orange and pink) and median values (black dots)

Table 1 shows a summary of the included participants’ age, sex, and cognitive functioning (verbal and non-verbal IQ). Two-sample independent-means t-tests were run in R [40] to test for group differences in age and cognitive performance. In cases in which the assumption of the homogeneity of variances was violated, *Welch* corrections were applied to adjust the degrees of freedom. A chi-square test was run to test for independence between sex and group. Effect sizes were calculated utilizing Cohen’s *d* and *w*. As can be seen in Table 1 and Fig. 1, the groups differed in verbal and non-verbal abilities, with individuals with cystinosis showing more difficulties than their age-matched peers. No differences were found in age and sex between the groups.

**Table 1:** Characterization of the control and cystinosis individuals included in the analyses: demographics and cognitive function (IQ and inhibition measures)

	Control	Cystinosis	Statistical test	Effect sizes
Age	M = 17.36; SD = 8.92	M = 17.62; SD = 9.36	*t* = −0.13, *df* = 75.43, *p* = 0.90	*d* = 0.03
Sex	27 F, 18 M	25 F, 12 M	χ^2^ = 0.23, *df* = 1, *p* = 0.63	w = 0.08
Verbal IQ	M = 109.71; SD = 19.85	M = 93.46; SD = 11.21	*t* = 4.66, *df* = 71.57, *p* < 0.01	*d* = 1.00
Non-verbal IQ	M = 103.44; SD = 13.49	M = 85.19; SD = 13.70	*t* = 6.04, *df* = 76.50, *p* < 0.01	*d* = 1.36

### Basic visual processing: P1

**Fig. 2:**
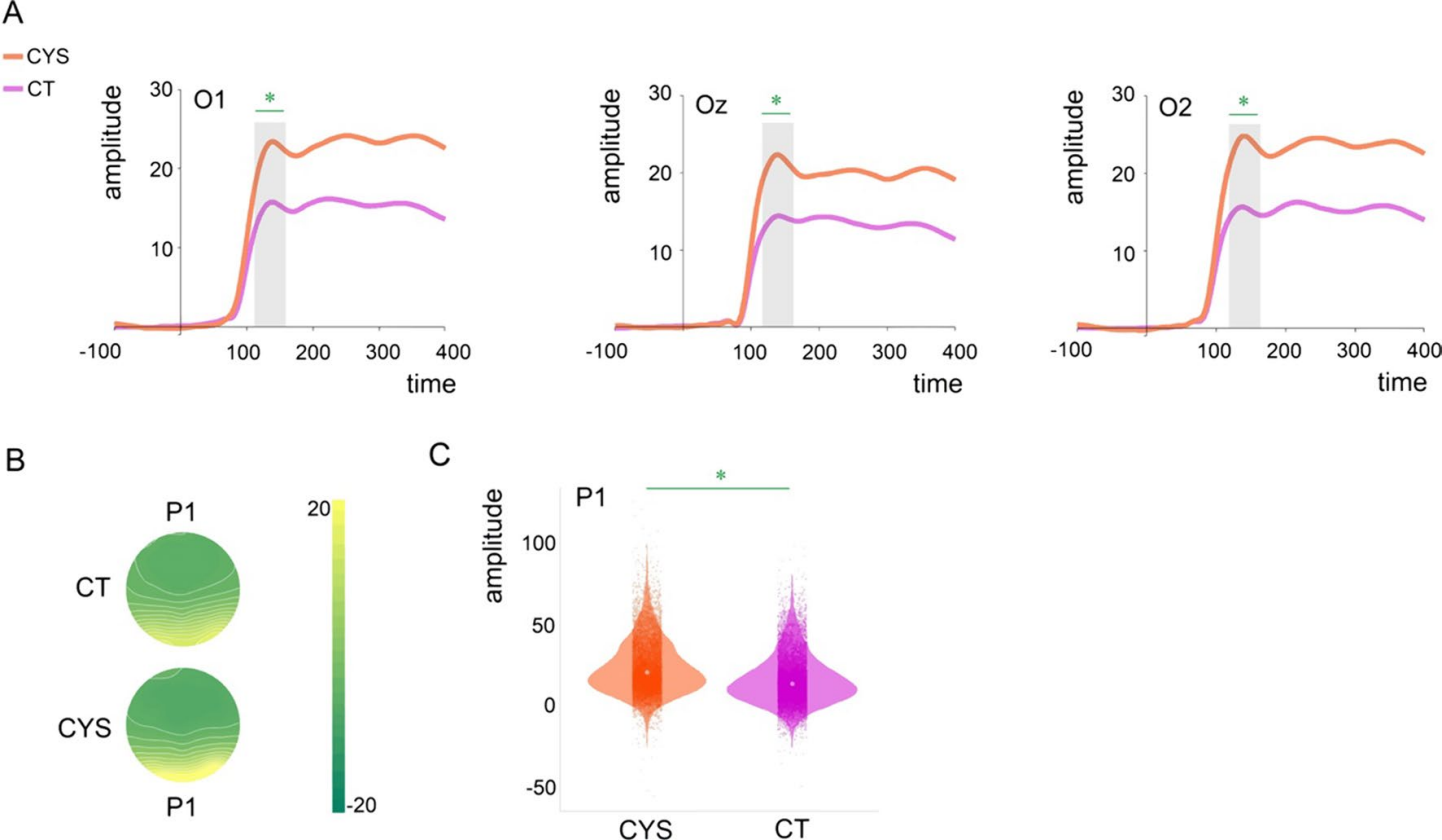
**A** Averaged ERPs per group and channel (O1, Oz, and O2). Shaded areas indicate window of interest. Asterisks indicate significant differences. **B** Topographical maps showing brain activity in the P1 time window per group. **C** Violin plots showing distribution of single trial data amplitudes per group at O1, Oz, and O2 (average) for P1. Small dots indicate amplitude at a given trial and central dots group mean amplitude. Asterisks indicate significant differences

The averaged VEPs for P1 per channel and by group can be seen in Fig. 2. Mixed-effects models were implemented as described in the Methods Section. Average amplitude at O1-Oz-O2 at each trial was the numeric dependent variable. As can be appreciated in the waveforms and scalp topographic maps in Fig. 2, individuals with cystinosis showed significantly increased P1 amplitudes compared to their age-matched peers (*ß* = 7.55, SE = 2.45, *p* = 0.01).

### Correlations

**Fig. 3:**
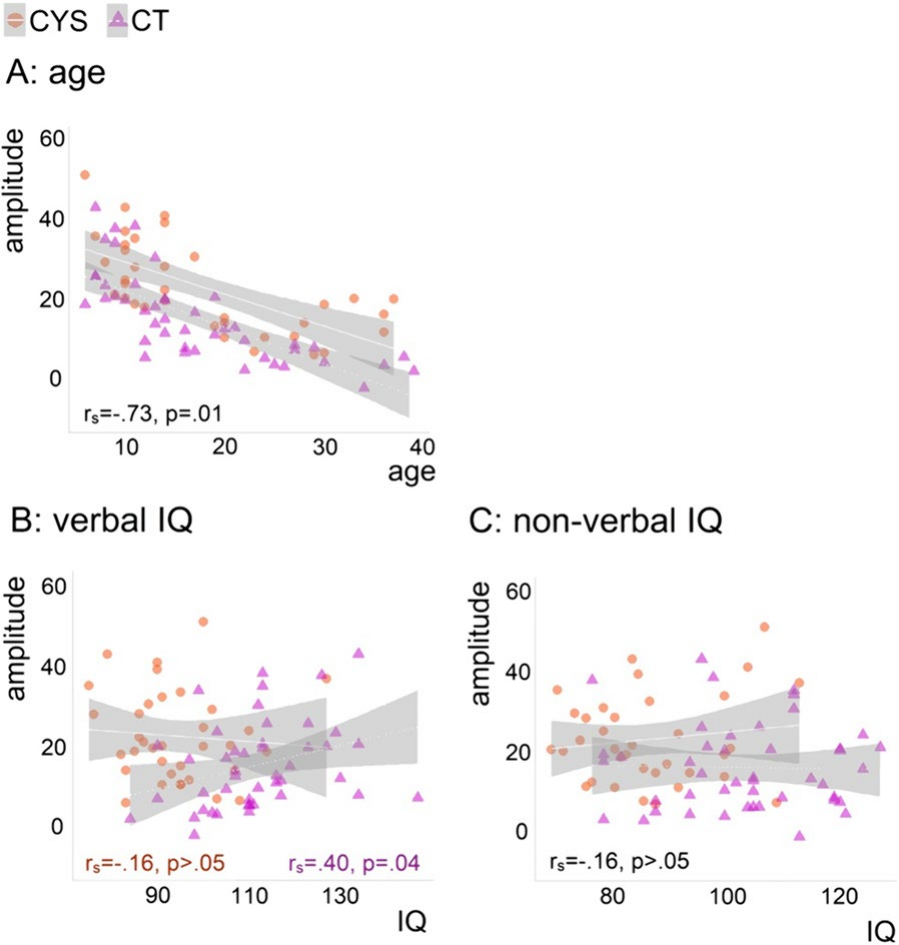
Spearman correlations between P1 and age (**A**), verbal IQ (**B**), and non-verbal IQ (**C**). Correlation coefficients and their significance are presented for the full sample when groups did not differ (age and non-verbal IQ) and per group when groups differed (verbal IQ)

Figure 3 shows neural response correlations with age (Panel A), verbal IQ (Panel B), and non-verbal IQ (Panel C). P1significantly correlated with age in both groups, with amplitudes decreasing with age (*r*_*s*_ = −0.73, *p* = 0.01). As can be seen in Fig. 3 Panel B, the groups differed in the correlations between P1 and verbal IQ. While controls showed a positive correlation between P1 and verbal IQ (*r*_*s*_ = 0.40, *p* = 0.04), such correlation was not significant in cystinosis. P1 did not correlate significantly with non-verbal IQ, for either the control or the cystinosis group.

## Discussion

Despite ample evidence for atypical visual processing in cystinosis in the neuropsychological literature [20, 26, 42] and clinical reports of hyper-sensitivity to visual stimulation, the integrity of basic visual sensory processing in this group has not been extensively explored. To begin to address this knowledge gap, we used scalp recordings of electrophysiological responses to visual stimuli to compare the amplitude of the visual P1, the earliest major neurophysiological response that was detectable in our data, between a group of individuals with cystinosis and an age matched control group. This revealed that in the individuals with cystinosis, the amplitude of the P1 was markedly amplified.

Increased sensory-evoked potentials may reflect increased excitability in sensory cortices [43–47]. An increased P1 in cystinosis could thus signify visual cortex hyperexcitability in this population. Visual cortex hyperexcitability has been linked to photophobia in individuals suffering from migraines [43], who may also present with increased visual evoked potentials (such as the P1)—though not consistently so (see [44] for a summary of different results). Importantly, photophobia is the most frequently reported ocular symptom in cystinosis [3, 48, 49]. Though we did not quantify photophobia in our cystinosis sample, comments regarding general sensitivity to light were common during testing. The pathophysiology of photophobia in relation to crystal accumulation in cystinosis is unclear. Our finding of a significantly increased visual evoked response in cystinosis opens the possibility that hypersensitivity to light could be related to altered visual processing due to visual cortex hyperexcitability.

Although we think it unlikely, one cannot rule out the possibility that group differences in P1 amplitude, alternatively, reflect different levels of attentional engagement. Selective attention, most often in the context of visuo-spatial attention designs, has been shown to significantly modulate P1 amplitude [50–56]. Such modulation may reflect a sensory gain type mechanism that results in enhanced perceptual processing of attended stimuli [57]. However, it is important to point out that attentional modulations of the P1 are typically only reported for stimuli that are presented peripherally (i.e. inputs that are not foveated) during selective covert spatial attention tasks, and a number of studies have shown that experimental manipulations of attention in the context of foveally presented stimuli do not induce P1 modulations (e.g., [58, 59]) or that it requires special manipulation of the nature of the centrally presented inputs [60]. Interestingly, we have previously reported increased P2 and P3a amplitudes in a sample of adults with cystinosis in the context of a passive auditory duration oddball stimulation [15]. We argued then that individuals with cystinosis may engage attention differently, which the current findings could be further evidence of. Studies focused on attentional processes in this population are needed, particularly those that relate to potential differences in attention engagement.

P1 has been localized to sources in the dorsal extrastriate cortex, specifically to areas V3, V3a, and adjacent middle occipital gyrus and in the ventral extrastriate cortex, specifically area V4 in the fusiform gyrus [29–32] and it is thus driven by both magnocellular and parvocellular input [61]. Traditionally, V3 and V3a have been associated with motion processing (e.g., [62]). However, studies in non-human primates suggest that these areas may also be involved in linking higher-level parietal and temporal processing streams [63] and may play a role in the integration of visual stimulus features, essential for global stimulus processing [64]. V4 has been associated with the processing of surface properties (color, brightness, texture), shape (orientation, curvature), motion and motion contrast, and depth; but it has also been shown to play a crucial role in visual attention [65]. Moreover, in non-human primates, V4 appears to be widely interconnected with other visual areas along the ventral and dorsal visual streams, frontal areas, and subcortical structures, and thus has been conceived as holding an integrative role in visual perception and recognition and, potentially, in guiding perceptual decisions and higher-order behavior [66]. Structural and/or functional neuroimaging studies examining V3, V3a, and V4 could be particularly informative in understanding the mechanistic roles that these areas play in the visual-spatial and visual attention differences described in cystinosis.

The increased amplitude VEP in cystinosis appears to be especially localized to the occipital and lateral parietal-occipital channels. While the current analyses focused on the channels in which response was maximal for both groups, those channels also represented the largest differences between the groups. As one can appreciate in Additional file 1: Figure S1, differences between the groups are reduced in lateral parietal-occipital channels and absent in mid-line parietal-occipital and across parietal channels. Future studies directed at defining the underlying neural sources explaining these differences are justified.

Correlational analyses revealed that neural indices of visual sensory processing correlated with age in a similar fashion across groups. In the general population, VEPs attenuate in amplitude through at least late adolescence (Brandwein et al. 2011). Here, P1 attenuation was replicated in our control sample and was also observed in the cystinosis group. The groups differed, however, in the correlations between P1 and verbal IQ. While controls with higher IQ scores showed larger P1s, a significant relationship between P1 and verbal IQ was not present in the cystinosis group. P1 did not correlate significantly with non-verbal IQ, for either the control or the cystinosis group.

Lastly, a brief detailing of findings relating to general cognitive function in cystinosis is merited. Neurocognitive assessments showed lower verbal and non-verbal IQs in individuals with cystinosis, when compared to their control peers. We and others had previously reported lower IQ scores in this population [15, 16, 19, 23] and, as in other studies, our findings (see Table 1) indicate greater difficulties in non-verbal processing [18–22, 27]. Of note, scales like the Wechsler Scales of Intelligence have an unbalanced number of timed non-verbal vs verbal subtests. To more accurately characterize the cognitive profile associated with cystinosis, it would be important to investigate whether, given the time, individuals with cystinosis would still show marked difficulties in non-verbal tasks or whether such difficulties are mainly explained by processing speed differences. While this would complicate comparisons with normative data, it could be useful to understand discrepancies between verbal versus non-verbal subtests.

This study is not without limitations. First, our groups were not matched in terms of IQ, with the cystinosis group presenting significantly lower scores than the control group. Although there is no evidence of the association between VEPs and IQ, such differences could have impacted the results. Second, variables related to current health status (such as a measure of renal function) and compliance with treatment, which has been linked to better clinical outcomes [67], were not included in the present study but could be useful in understanding group- and individual-level differences. Lastly, most of the individuals with cystinosis, and differently from those included in the control group, travelled to the lab from out-of-state and completed the study across two consecutive days, which could have increased tiredness in this group.

To conclude, here we present evidence of visual processing differences in cystinosis which could contribute, at least partially, to both photosensitivity and to the behavioral visual-spatial difficulties described in this population. More work is needed to describe how visual processing differences might contribute to the cognitive profile associated with cystinosis. A better understanding of such associations could contribute to the identification of sensitive biomarkers of treatment efficacy on brain function. The current findings should motivate future studies utilizing paradigms tapping into higher- and lower-order visual processes and investigating visual processing and pathways in more depth, for instance, by employing tasks distinguishing between ventral and dorsal streams and magnocellular and parvocellular pathways. However, in so doing we believe it is important for future EEG research with this population to take sensitivity to light into consideration when designing visual experiments. Furthermore, these findings and their implications in classroom and professional settings utilizing digital media and screens should be further explored.

### Supplementary Information

**Additional file 1.** Averaged ERPs per group over parietal, parietal-occipital, and occipital channels.

## Data Availability

All data are available from the corresponding authors on reasonable request.

## Abbreviations

EEG: Electrophysiological
VEP: Visual-evoked potential
CT: Controls
CYS: Cystinosis
