# Supplementary material for: Event-related potential (ERP) evidence for visual processing differences in children and adults with cystinosis (CTNS gene mutations)
Source: Orphanet J Rare Dis. 2023 Dec 12;18:389. doi: 10.1186/s13023-023-02985-y (PMC10714457; doi:10.1186/s13023-023-02985-y)
Supplement: Supplementary file 1 — Additional file 1. Averaged ERPs per group over parietal, parietal-occipital, and occipital channels. [file 13023_2023_2985_MOESM1_ESM.docx]

**SUPPLEMENTARY MATERIALS**


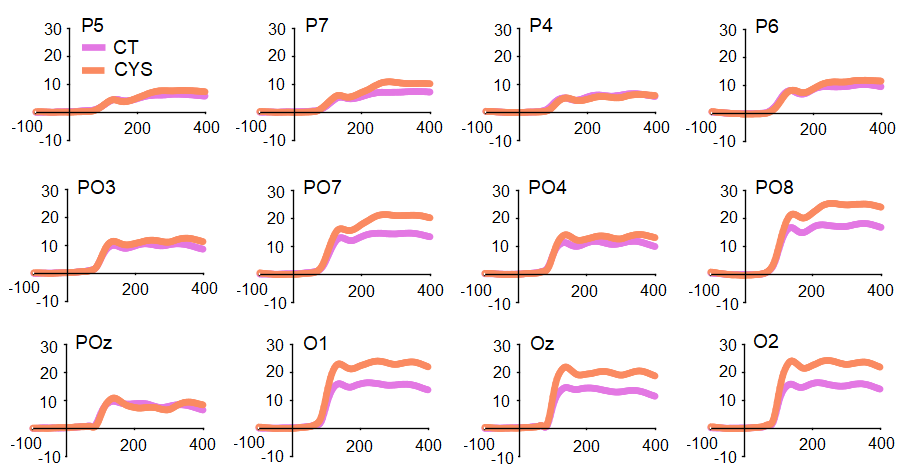


Figure S1. Averaged ERPs per group over parietal, parietal-occipital, and occipital channels.
